# Clinical Case of a 23-Year-Old Patient with Moyamoya Disease and Epilepsy in Bulgaria

**DOI:** 10.3390/neurolint16040065

**Published:** 2024-08-20

**Authors:** Ekaterina Viteva, Petar Vasilev, Georgi Vasilev, Kostadin Chompalov

**Affiliations:** 1 Department of Neurology, Faculty of Medicine, Medical University of Plovdiv, 4000 Plovdiv, Bulgaria; eiviteva@abv.bg (E.V.); petarvasilev1996@gmail.com (P.V.); kostadinchompalov@mail.bg (K.C.); 2 Clinic of Neurology, UMHAT “Sv. Georgi”, 4000 Plovdiv, Bulgaria; 3 Medical Faculty, Sofia University St. Kliment Ohridski, 1 Kozyak Str., 1407 Sofia, Bulgaria

**Keywords:** cerebral vasculopathy, moyamoya, ischemic stroke in young age, transient ischemic attack

## Abstract

Moyamoya disease is a cerebrovascular pathology characterized by progressive stenosis of the internal carotid arteries and their branches, leading to ischemic and/or hemorrhagic disorders of the cerebral circulation, primarily affecting children and young adults. We present a case of a 23-year-old woman with a history of recurrent cerebrovascular accidents since childhood. Despite experiencing focal motor seizures and transient ischemic attacks, her condition remained undiagnosed until 2006, when, at the age of 7, a digital subtraction angiography revealed characteristic bilateral internal carotid artery occlusions. Subsequent diagnostic challenges and treatments preceded a worsening of symptoms in adulthood, including generalized tonic–clonic seizures. Upon presentation to our clinic, the patient exhibited upper motor neuron syndrome and occipital lobe syndrome, consistent with the disease’s pathophysiology, neuroimaging, and clinical manifestations. Imaging studies confirmed multiple ischemic lesions throughout the cerebral vasculature. Treatment adjustments were made due to the increased incidence of seizures, and the dose of her anti-seizure medication—divalproex sodium—was increased. This case underscores the diagnostic complexities and challenges in managing moyamoya disease, emphasizing the importance of early recognition and prompt intervention.

## 1. Introduction

Moyamoya disease and the associated moyamoya syndrome represent a cerebrovascular pathology entity characterized by progressive stenosis of the internal carotid arteries and their proximal branches and ischemic and/or hemorrhagic disorders of the cerebral circulation beginning at a young age [1].

**Figure 1:**
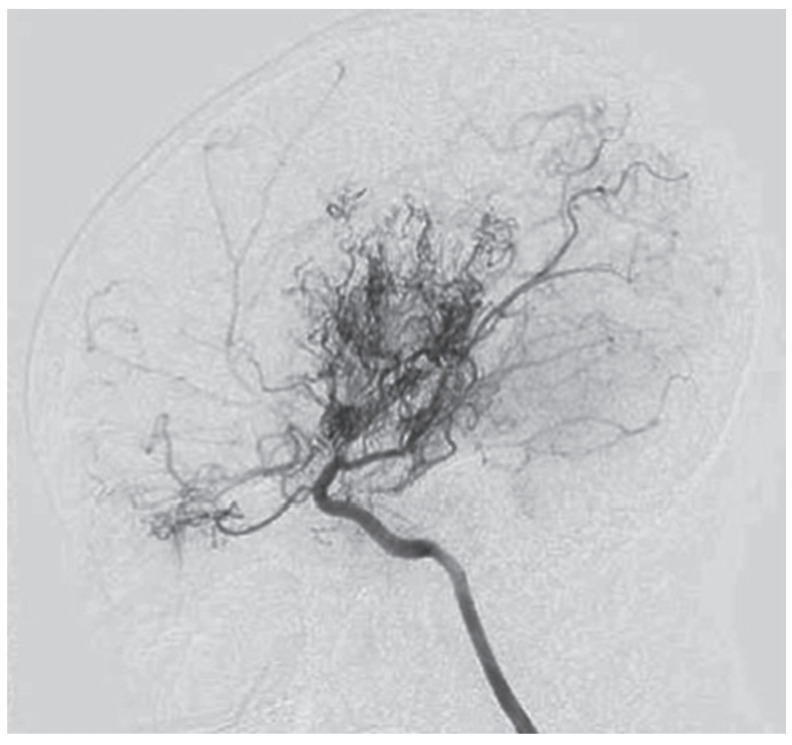
Angiographic findings in moyamoya [1].

The first description of this condition dates back to 1957, when it was termed “hypoplasia of the bilateral internal carotid arteries”. Following the progressive narrowing of blood vessels of the anterior circulation, a dense collateral network of new vessels develops near the carotid apex, the cortical surface of the cerebral hemispheres, the leptomeninges, and around branches of the external carotid artery, supplying the dura mater and the skull base. Rarely, such a network may form among the vessels of the posterior circulation, namely the basilar artery and posterior cerebral arteries. The characteristic appearance of those newly formed and dilated vessels on conventional angiography was described as “hazy, like a puff of cigarette smoke” (Figure 1), which in Japanese translates to moyamoya [1,2].

Even though a contemporary term has recently been proposed for the diagnosis, namely “spontaneous occlusion of the circle of Willis”, the International Classification of Diseases recognizes it under the name “moyamoya” [3].

## 2. Clinical Case Description—Past Medical History of Our Patient

A 23-year-old woman presented to our clinic with an established diagnosis of moyamoya disease since childhood and having suffered from numerous cerebrovascular accidents since then. Her medical history began in 2004, at the age of 5, when during a stressful traumatic event, she experienced a focal motor tonic seizure of the left limbs and the left half of the face. She continued to experience such events with a frequency of 2 to 3 times monthly, mainly associated with emotional stress. No diagnostic studies and treatment were undertaken, and the seizures were attributed to being “psychogenic” in origin. After a brief period of improvement, the seizures re-emerged, and new focal seizures with weakness and paresthesia affecting the right limbs, as well as dysarthria, occurred with a similar frequency and with a duration of about 10–15 min. An EEG at that time showed bilateral frontotemporal slow-wave activity, more pronounced on the right. Magnetic resonance imaging performed in 2005 demonstrated significant cortical atrophy affecting the right hemisphere and a suspicion of the syndrome of Sturge–Weber was raised. The seizures continued to occur; however, no further studies and specialist consultations were performed.

Then, in the summer of 2006, one morning, the child was abruptly unable to speak and her right limbs were weak. The motor deficit resolved over the course of 2 to 3 h, while the ability to speak resolved later, after 24 h. The child was then hospitalized in a pediatric neurosurgical facility with residual right arm weakness. A digital subtraction angiography was performed, which showed bilaterally absent contrast in both the anterior and middle cerebral arteries due to significant occlusions in the intracranial parts of both internal carotid arteries. A fine vascular network was visualized around the bifurcations of the internal carotids, as well as, to a lesser extent, around the basilar and posterior cerebral arteries. Immediately after the angiography, the child was somnolent and febrile, and her neurological status deteriorated, with right-sided facial palsy, right-sided hemiplegia, and total aphasia. It was hypothesized that this complication occurred due to a vasospasm during the procedure. The complete blood count, basic metabolic panels, inflammatory markers, and chest X-ray were all unremarkable. In the following hours and days, the child’s condition slowly improved, though not fully, and she was discharged with right-sided facial palsy and partial motor aphasia. It was presumed that cerebral vasculitis may have been the culprit, leading to diffuse and focal cerebral ischemia. However, the characteristic appearance of the angiographic findings was deemed enough evidence to establish the diagnosis of moyamoya disease.

**Figure 2:**
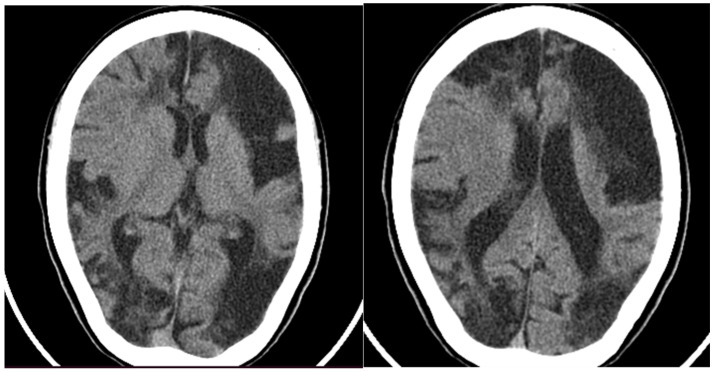
CT of the head showing large hypodense lesions in the (**left**) hemisphere and smaller ones on the (**right**).

Later the same year, the child was hospitalized in a pediatric neurology department, where a CT of the head was performed (Figure 2), showing large hypodense areas with cerebrospinal fluid density, predominantly in the left hemisphere, smaller hypodense areas in the right hemisphere, and ventriculomegaly. A screening panel for autoimmune disorders was performed, including anticardiolipin antibodies, rheumatoid factor, antinuclear antibodies (ANA), ds-DNA antibodies, anti-Smith antibodies, anti-Ro (SS-A) and anti-La (SS-B) antibodies, and p- and c-ANCA- antibodies, with only the VDRL anti-cardiolipin antibodies testing slightly positive. Furthermore, mitochondrial disease screening revealed a mutation in the MT-ND2 gene, which was not reported to be pathogenic. At that time, treatment with pentoxifylline and piracetam was initiated, leading to a temporary improvement in weakness and speech; no new emergence of cerebrovascular symptoms occurred.

At the beginning of 2008, the child complained of impaired vision, with an ability to see only nearby objects centrally, pulsating left frontal headache, inability to articulate words, and inability to swallow food and saliva. She was admitted again to the same pediatric neurology department. Neurologic examination revealed “tunnel” vision with bitemporal hemianopia, quadriparesis with more pronounced weakness of the right limbs, slightly increased muscle tone in the distal regions of all limbs, more on the right, flexion contracture of the right hand, pathologically increased deep tendon reflexes on the right, with pathological reflexes of Babinski and Tromner present on the right, clonus of the right foot, right-sided hemisensory deficit, and partial motor aphasia. CT of the head showed no new findings compared to the one from 2006. EEG found pronounced slow-wave activity in the right hemisphere and slowed activity in the left hemisphere, with no prominent paroxysmal changes. During the hyperventilation test, the child once again exhibited a pulsating frontal left-sided headache, worsening of the present dysarthria, and a temporary right-sided central facial palsy. It has been well established in clinical studies that hyperventilation exacerbates the symptoms of moyamoya disease and so it should be avoided [4]. The present bitemporal hemianopia was re-evaluated and was hypothesized to represent bilateral macula-sparing hemianopia due to lesions of the optic tracts and the cortical visual fields. Based on history, neurologic findings, and laboratory studies, the seizures were attributed to transient ischemic attacks with no epileptic origin. Low-dose aspirin at night was added to the therapy.

## 3. Onset of Epileptic Seizures

Years later, in 2019, our patient began experiencing generalized tonic–clonic seizures, predominantly during the night. In the beginning, the frequency was 2–3 seizures per month. After a consultation with an outpatient neurologist, treatment with divalproex sodium 2 × 500 mg daily was prescribed.

## 4. Current Hospitalization in Our Clinic

In the summer of 2023, at the age of 23, the patient was hospitalized in our clinic for treatment assessment. Until that time, the patient had had a total of 12 seizures since 2019. The physical examination was unremarkable, except for an asthenic overall habitus and chronic venous insufficiency of the lower limbs. She was alert and oriented to person, time, and place, although mildly cognitively impaired. Neurologic examination revealed the following:

1. Upper motor neuron syndrome: hyper-reflexia for the deep tendon reflex (right > left), patellar clonus on the right, marked distal right arm weakness and slight weakness of the left leg, reflex of Babinski present on the right;
2. Occipital lobe syndrome: prosopagnosia and object agnosia.

**Figure 3:**
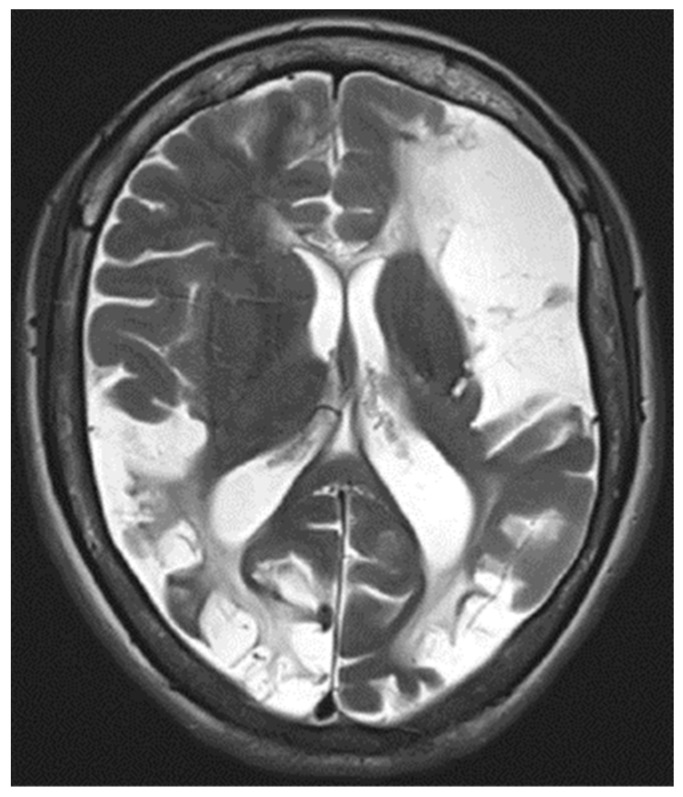
T2-weighted MRI image showing diffuse ischemic lesion throughout the cerebral hemispheres.

**Figure 4:**
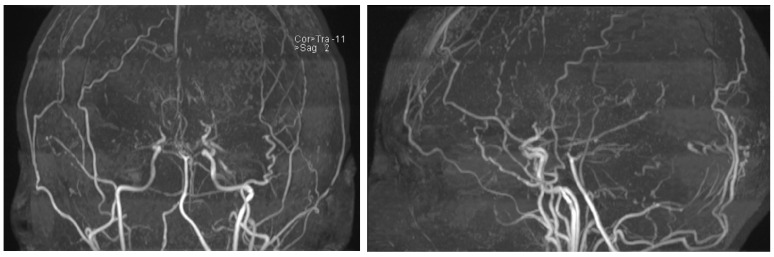
MRI time-of-flight (TOF) angiography showing significantly diminished blood flow in the anterior circulation and the development of collateral blood vessels.

An MRI of the head (Figure 3) showed multiple long-standing ischemic lesions in all regions of the cerebral vasculature and significantly reduced blood flow in all arteries of the anterior circulation (Figure 4). Doppler ultrasonography of the extracranial vessels did not show any abnormalities. EEG revealed marked interhemispheric asymmetry—diffusely slowed, not well-organized brain activity in the right hemisphere, and in the left, not well-organized alpha rhythm with frequent single or multiple temporoparietal spikes. Abdominal ultrasound, consultation with a vascular surgeon, duplex ultrasonography of the lower limb vessels, consultation with a cardiologist, ECG, and echocardiography were all unremarkable for any pathology. Computed perimetry for the evaluation of visual field defects could not be accomplished due to excessive myopia, reduced visual acuity (20/800), and cognitive impairment. The serum level of valproate was 61.68 μg/mL.

**Figure 5:**
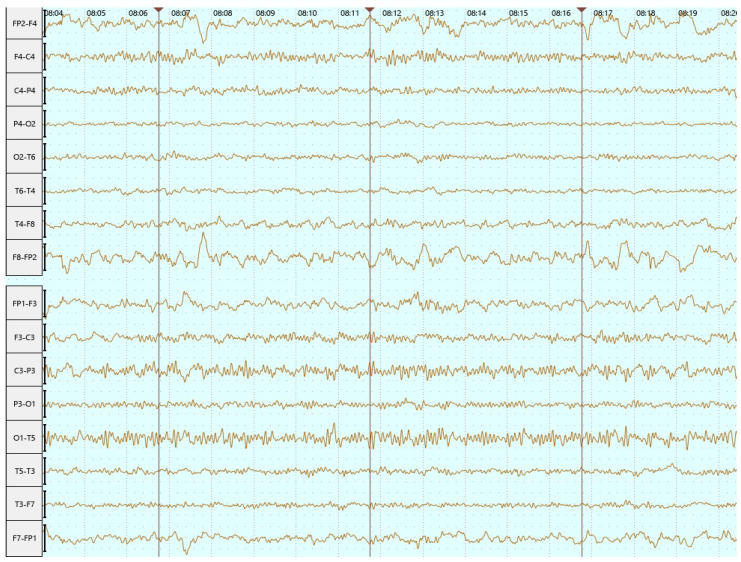
EEG in June 2023 (bipolar longitudinal montage)—abnormal EEG with interhemispheric asymmetry and focal left temporoparietal irritative changes.

Based on the EEG finding (Figure 5) and persistent paroxysmal symptoms, a decision was made to increase the dose of divalproex sodium to 3 × 500 mg daily. Six months after hospitalization, the patient had not had any seizures, making it a total of 11 months without seizures.

## 5. Neuropsychological Assessment

During a recent follow-up visit to our clinic, an extensive neuropsychological assessment was undertaken. The patient has had 12 years of formal education, having attended a school for visually impaired children and adolescents. She is right-handed. There were no reports of current or past psychiatric problems, either in the patient or in other family members. In everyday life, she is independent to a large extent, although she is limited by her reduced vision and weakness of the right hand. Only central vision is preserved to an extent that allows her to function in a normal environment. When necessary for manipulation and writing, she uses her left hand. Her current general intellectual functioning corresponds to mild cognitive impairment (MMSE = 26 points). Orientation to her own person, time, and place is preserved. Attention/concentration is slightly impaired. She exhibits difficulty with arithmetic skills (subtracting 7 from 100). Language function/speech is preserved at the social level. She makes spelling mistakes when writing sentences spontaneously. Repeated speech is difficult, especially for less frequently used words and in more complex sentences. She shows a naming deficit. Visuospatial skills are most negatively affected. The patient shows an inability to visually recognize and redraw two interlocking pentagons, some numbers and printed letters, and realistic shapes of everyday objects. She does not recognize the faces of close people, but names them after seeing the rest of the body. Anterograde verbal memory is preserved. When trying to remember words and combinations of words with less frequent and specific use, repetition is needed.

As a conclusion of the neuropsychological assessment, the patient was diagnosed with a mild neurocognitive disorder, with a deficit in the sphere of attention, working memory, speed of information processing, and a dominance of disorders in visual–spatial function. Based on the data obtained, the patient’s current condition corresponds to apperceptive agnosia and prosopagnosia [5,6]. These defects also correspond to the lesions found on brain neuroimaging. It should be noted that gnosis is formed through education, and the patient’s vision disturbance occurred at an early school age. Therefore, it is reasonable to assume that agnostic manifestations are also partially related to lack of academic knowledge and skills, as well as to problems in the use of the alphabet and spelling [7]. From a therapeutic point of view, the following are recommended in this case: cognitive rehabilitation therapy, focused on visual–spatial skills and object recognition; regular monitoring and assessment of cognitive status to monitor symptom progression; speech therapy to improve language skills and object naming [8,9]. The results of the neuropsychological examination emphasize the need for follow-up therapy and regular monitoring of the patient’s cognitive functions to prevent further cognitive and functional impairment.

## 6. Discussion

Moyamoya disease is an increasingly recognized cause of ischemic and/or hemorrhagic cerebrovascular disease in children and young to middle-aged adults. Its occurrence reaches its highest points within two age groups: around 5 years of age for children and in the mid-40s for adults. Female patients outnumber males nearly twice. Moyamoya stands as the predominant cerebrovascular disease among children in Japan, affecting roughly 3 out of every 100,000 children [10]. In Europe, the incidence among all moyamoya patients seems to be approximately one-fifth to one-tenth of the rate observed in Japan [11]. In a study titled “Moyamoya Disease in Europeans,” Kraemer and colleagues reported on the incidence and prevalence of moyamoya disease in European populations. They found that moyamoya disease is significantly rarer in Europe compared to East Asian countries. The estimated incidence in Europe was about 0.06 per 100,000 individuals per year, which is much lower than in Japan, where the incidence is approximately 0.35 per 100,000 individuals per year [12].

The European Stroke Organization (ESO) guidelines on the diagnosis and management of moyamoya disease also mention that while the disease is well documented in Asia, it remains a rare condition in Europe. They cite an incidence rate similar to Kraemer’s findings and emphasize the need for increased awareness and specialized diagnostic approaches in European medical practice [13].

These sources provide robust evidence that moyamoya disease is rare in Europe, with significantly lower incidence and prevalence rates compared to East Asia. This rarity underlines the importance of reporting unique cases and experiences with the disease in European populations to contribute to the global understanding and management of moyamoya disease.

Moyamoya disease can present with intracranial hemorrhage in some patients. A cohort study of 200 Caucasian patients with the disease estimated the proportion of those with hemorrhagic presentation to be 9.5% [14].

Certain conditions such as sickle cell disease, Down syndrome, neurofibromatosis 1, type 1 diabetes mellitus, and Graves’ disease are more commonly associated with the development of moyamoya disease [15,16,17]. Moyamoya is also closely associated with radiotherapy to the head or neck, especially that performed for conditions like optic gliomas, craniopharyngiomas, and pituitary adenomas. However, the specific radiation dose responsible for this effect remains unclear, and the onset of the disease can occur anywhere from months to decades after treatment [18].

Genetic factors are commonly suggested in moyamoya, with approximately 10% of patients in Japan and 6% in the U.S. having first-degree relatives affected by the disease. Familial cases often involve multiple genes or follow an autosomal dominant pattern with incomplete penetrance. A study in 2008 identified a major gene locus on chromosome 17q25 and a mutation associated with moyamoya in this region, affecting TIMP-2 (tissue inhibitor of matrix metalloproteinase type 2), which is noteworthy due to its role in extracellular matrix remodeling and angiogenesis [19]. Despite the genetic basis, cases of only one affected twin among identical twins suggest environmental factors may trigger the condition in susceptible individuals [20].

The RNF213 gene, especially the R4810K (p.R4757K) variant, is a key genetic factor associated with moyamoya disease (MMD). This single nucleotide polymorphism (SNP) is most prevalent in East Asian populations, with studies showing that around 80% of Japanese and Korean MMD patients carry this mutation. The RNF213 R4810K variant is highly prevalent among East Asian MMD patients but is much rarer in other populations. This variant is found in about 2% of the general East Asian population, indicating a strong genetic predisposition in these regions [21]. The RNF213 gene is involved in angiogenesis and vascular remodeling. Mutations like R4810K are believed to disrupt these processes, leading to the abnormal vascular networks seen in MMD. Although the precise mechanisms are still under study, this mutation is linked to impaired endothelial cell function and response to hypoxia, contributing to disease progression [22]. Although less common, RNF213 mutations are also found in non-Asian MMD patients, indicating its broader relevance. This suggests that while RNF213 is a critical factor, other genetic or environmental influences might also play a role in different ethnic groups. The identification of RNF213 variants, especially R4810K, has significant implications for early diagnosis and genetic counseling in MMD. Testing for these SNPs can help identify at-risk individuals, particularly in families with a history of MMD, aiding in early intervention and management [21].

The natural course of moyamoya varies. Progression may be slow and indolent, or it may be fulminant, leading to a rapid neurological decline. Nonetheless, the disease typically advances with time in most patients [1]. A 2005 report highlighted the high rate of progression, even in asymptomatic cases, with medical therapy alone unable to halt it [23]. Ultimately, the neurological status at the time of diagnosis and initiation of treatment, rather than the patient’s age, predicts long-term outcomes. Hence, early diagnosis and prompt therapy are crucial in managing moyamoya [24].

In regards to the frequency of epileptic seizures complicating the course of the disease, a study by Starke et al. (2009) reports that seizures were observed in about 10% of adult patients with moyamoya disease. The authors highlight that while ischemic and hemorrhagic events are more common, seizures are a notable neurological manifestation [25].

Another article published in 2009 by Scott and Smith in the *New England Journal of Medicine* mentions that epileptic seizures occur in approximately 5–10% of moyamoya patients. They discuss the pathophysiology behind the seizures, linking them to chronic cerebral ischemia and frequent strokes associated with moyamoya disease [1].

Epidemiological data by Guey et al. (2015) note that seizures are reported in about 8–10% of patients with moyamoya disease [26]. These sources collectively highlight that while seizures are not the most common symptom in moyamoya disease, they may occur in a notable percentage of patients, typically ranging from 5% to 10%.

Timely diagnosis is critical, especially in children presenting with acute neurological deficits or unexplained symptoms indicative of cerebral ischemia. Detecting and addressing moyamoya promptly is essential to minimize the risk of permanent disability. A lumbar puncture and suitable imaging modalities are of utmost importance in differentiating the condition from a CNS infection, a neoplasm or an arteriovenous malformation. Certain radiographic studies play a pivotal role in confirming the diagnosis, often necessitating several evaluations to capture the full extent of the condition [1].

CT scans may reveal small areas of hypodensity, indicating potential past microhemorrhages or ischemic strokes, particularly in regions like the cortical watershed zones, basal ganglia, deep white matter, or periventricular areas. However, it is worth noting that CT scans might appear normal, especially in cases of patients who only present with transient ischemic attacks (TIAs). CT angiography usually depicts the characteristic intracranial stenoses associated with moyamoya, making it a valuable tool when magnetic resonance imaging is not readily available. MRI and magnetic resonance angiography (MRA) have become increasingly utilized as primary imaging modalities for moyamoya. These techniques offer superior resolution and detail compared to CT scans. Additionally, reduced cortical blood flow, a hallmark of moyamoya, can be inferred from fluid-attenuated inversion recovery (FLAIR) sequences, which display linear high signals following a sulcal pattern, known as the “ivy sign”. Moreover, MRI can reveal diminished flow voids in the internal, middle, and anterior cerebral arteries coupled with prominent flow voids through the basal ganglia and thalamus from moyamoya-associated collateral vessels. This finding is unequivocally diagnostic of moyamoya disease [27].

Angiography, whether conventional or computed, is crucial for confirming the diagnosis definitively. A full five- or six-vessel study is typically performed, encompassing both internal and external carotid arteries, as well as vertebral arteries, to capture the complete vascular anatomy. In moyamoya, characteristic arteriography findings include stenosis of distal intracranial carotid arteries, often extending to the proximal anterior and middle cerebral arteries [1]. The Suzuki grading system aids in staging the severity of the disease in six stages, with the presence of an extensive collateral network (“puff of smoke”) indicative of intermediate stages [2]. Imaging of the external carotid arteries is vital to identify preexisting collateral vessels, ensuring surgical interventions do not disrupt critical blood flow pathways. Additionally, conventional angiography is essential for detecting any associated aneurysms or arteriovenous malformations, which can complicate the management of moyamoya [1]. CT-angiography (CTA) and CT-perfusion (CTP) are valuable tools in the diagnosis and management of moyamoya disease. CTA is particularly useful for visualizing the patency of grafts after surgical revascularization and ensuring the success of procedures like superficial temporal artery to middle cerebral artery (STA-MCA) bypass. CTP provides functional imaging that measures cerebral blood flow (CBF), cerebral blood volume (CBV), mean transit time (MTT), and time to peak (TTP). These parameters are essential for understanding the perfusion status of brain tissue affected by moyamoya disease. The technique involves dynamic scanning following the injection of contrast material, allowing for the creation of detailed perfusion maps identifying regions at risk for ischemic damage. These maps help in determining the extent of blood flow impairment and guide surgical planning by highlighting areas that would benefit from revascularization procedures. The combined use of CTA and CTP allows for a comprehensive assessment of both the structural and functional aspects of the cerebral vasculature in moyamoya disease [28].

So far, no treatment can target the primary pathophysiological processes in moyamoya disease and all proposed treatments aim to improve blood flow to the affected hemisphere, thereby reducing the risk of strokes and alleviating associated symptoms. Medical therapy, primarily used in cases where surgery poses high risks or when the disease is still at a mild stage, lacks substantial evidence of efficacy. Antiplatelet agents are commonly prescribed to prevent microthrombi formation, although its use should be judicious due to the potential of a hemorrhagic presentation of moyamoya disease [1,26]. According to the European Stroke Organization (ESO) guidelines, antiplatelet therapy is particularly emphasized in the non-surgical management of moyamoya disease. An antiplatelet agent is prescribed in two main scenarios—in patients that are not suitable for surgical treatment and in the peri-procedural period of revascularization techniques to reduce the risk of ischemic complications. Antiplatelet therapy may be prescribed for an extended period of the time postoperatively to improve long-term outcomes. Overall, the ESO guidelines suggests the use of single antiplatelet therapy in non-hemorrhagic moyamoya disease to reduce the risk of ischemic strokes [24]. Calcium channel blockers such as verapamil and amlodipine may help manage vasospasms and headaches. They might also be able to reduce the severity and frequency of TIAs [1,26].

The external carotid artery is usually spared in moyamoya. Surgical interventions, which utilize the external carotid artery to provide alternative blood flow to the ischemic area, are accomplished by two approaches: direct and indirect revascularization. Direct procedures are often preferred in adults for immediate benefits, while indirect techniques stimulate neoangiogenesis over time, making them suitable for children [28,29]. Both methods have shown success in different cases, with some centers advocating a combination of both. Indirect revascularization techniques include various procedures like pial synangiosis, which have demonstrated significant stroke reduction rates post-surgery [27]. The 2023 ESO guidelines on Moyamoya angiopathy endorsed by Vascular European Reference Network (VASCERN) recommend only direct bypass surgery on adults with hemorrhagic presentation, and for ischemic adult patients and children, it was suggested that revascularization surgery using direct or combined technique should be prioritized rather than indirect methods alone, with an interval of 6–12 weeks between the last cerebrovascular event and surgery [28,30].

Surgical revascularization is increasingly accepted as the primary treatment for moyamoya, given its superior outcomes compared to medical therapy [30,31,32]. It carries some risks, including ischemic stroke, infection, and intracranial bleeding, especially in the perioperative period [33]. In cases of acute symptoms, immediate measures to enhance cerebral blood flow, such as adequate hydration with 1.5 to 2 times the maintenance doses of intravenous crystalloid solutions, avoidance of hypotension, and supporting ventilation and oxygenation, are crucial to prevent the progression of a transient ischemic attack to a completed stroke. Following the exclusion of intracranial hemorrhage by neuroimaging, aspirin is administered at a dose of 325 mg for adults and 81 mg or less for preteen children [1].

## 7. Conclusions

This case is of clinical interest due to the low frequency of moyamoya disease in Europe, pronounced brain parenchymal changes, and epileptic seizures. Particularly in Bulgaria, this is the first case report to describe the condition, highlighting its rarity in our region. We would like to emphasize the diagnostic challenges that this condition may and to underscore the treatment modalities available if recognized early in its course.

## Data Availability

More detailed data on the patient’s case report is available upon request by the authors.
